# Delayed educational reminders for long-term medication adherence in ST-elevation myocardial infarction (DERLA-STEMI): Protocol for a pragmatic, cluster-randomized controlled trial

**DOI:** 10.1186/1748-5908-7-54

**Published:** 2012-06-09

**Authors:** Noah M Ivers, Jon-David Schwalm, Jeremy M Grimshaw, Holly Witteman, Monica Taljaard, Merrick Zwarenstein, Madhu K Natarajan

**Affiliations:** 1 Family Practice Health Centre, Women’s College Research Institute, Women’s College Hospital, 76 Grenville Ave Toronto, Toronto, Ontario, Canada; 2 Department of Medicine, Division of Cardiology, McMaster University/Hamilton Health Sciences, 237 Barton Street, East Hamilton, Ontario, Canada; 3 Ottawa Hospital - General Campus, Centre for Practice-Changing Research, 501 Smyth Road, Ottawa, Ontario, Canada; 4 Center for Bioethics & Social Sciences in Medicine, Department of Internal Medicine, University of Michigan, 300 North Ingalls Ann Arbor, Michigan, USA; 5 Ottawa Hospital Research Institute, Clinical Epidemiology Program, Ottawa Hospital, Civic Campus, 1053 Carling Avenue, Ottawa, Ontario, Canada; 6 Institute for Clinical Evaluative Sciences, Sunnybrook Health Sciences Centre, 2075 Bayview Avenue, Toronto, Ontario, Canada

**Keywords:** Randomized trial, Medication adherence, Reminders

## Abstract

****Background**:**

Despite evidence-based recommendations supporting long-term use of cardiac medications in patients post ST-elevation myocardial infarction, adherence is known to decline over time. Discontinuation of cardiac medications in such patients is associated with increased mortality.

****Methods/design**:**

This is a pragmatic, cluster-randomized controlled trial with blinded outcome assessment and embedded qualitative process evaluation. Patients from one health region in Ontario, Canada who undergo a coronary angiogram during their admission for ST-elevation myocardial infarction and who survive their initial hospitalization will be included. Allocation of eligible patients to intervention or usual care will take place within one week after the angiogram using a computer-generated random sequence. To avoid treatment contamination, patients treated by the same family physician will be allocated to the same study arm. The intervention consists of recurrent, personalized, paper-based educational messages and reminders sent via post on behalf of the interventional cardiologist to the patient, family physician, and pharmacist urging long-term adherence to secondary prevention medications. The primary outcome is the proportion of patients who report in a phone interview taking all relevant classes of cardiac medications at twelve months. Secondary outcomes to be measured at three and twelve months include proportions of patients who report: actively taking each cardiac medication class of interest (item-by-item); stopping medications due to side effects; taking one or two or three medication classes concurrently; a perfect Morisky Medication Adherence Score for cardiac medication compliance; and having a discussion with their family physician about long-term adherence to cardiac medications. Self-reported measures of adherence will be validated using administrative data for prescriptions filled.

****Discussion**:**

This intervention is designed to be easily generalizable. If effective, it could be implemented broadly. If it does not change medication utilization, the process evaluation will offer insights regarding how such an intervention could be optimized in future.

****Trial registration**:**

Clinicaltrials.gov NCT01325116

## Background

### **Cardiovascular disease burden and the role for long-term pharmacotherapy**

Worldwide, cardiovascular disease (CVD) is estimated to be the leading cause of death and disability [1]. Approximately 50% of myocardial infarctions (MIs) and 70% of CVD deaths occur in patients who have already documented coronary artery disease (CAD) [2]. Therefore, the prompt identification of modifiable cardiovascular risk factors and initiation of proven secondary preventative medications post-MI are essential to the prevention of subsequent cardiac events [3]. Population-level observational studies provide evidence that the rate of cardiovascular morbidity and mortality has been decreased through the use evidence-based therapies [4,5].

ST-segment elevation myocardial infarction (STEMI) is a common presentation of acute myocardial infarction (AMI) constituting approximately 30% of all cases [6]. Post-STEMI, patients are at high risk for subsequent cardiac events—18% of men and 35% of women will have a repeat MI within six years and STEMI patients have four to six times the risk of sudden cardiac death compared to the general population [7]. While acute treatment is crucial for STEMI patients, relevant guidelines emphasize that the initiation and long-term maintenance of evidence-based secondary preventative therapies are essential for reducing the overall burden of CVD [3,8,9].

### **Poor long-term adherence to cardiac medications**

While there is a significant body of evidence supporting these guidelines (Table 1), there remains a large gap between ideal and actual care with regard to the long-term management of cardiovascular risk for these patients. Studies show that adherence to evidence-based therapies begins decreasing at 30 days and falls to as low as 50% adherence at six months post-discharge [10-14]. Unfortunately, discontinuation of evidence-based therapies has repeatedly been shown to be associated with increased mortality in patients with CAD [15-18].

**Table 1:** Summary of guideline recommendations for medications post-STEMI

**Medication**	**Recommendation**		Strength of evidence*
Anti-platelets	Aspirin therapy (75-162 mg/day) indefinitely post-STEMI.		I A
	P2Y_12_-receptor inhibitor (clopidogrel, prasugrel, or ticagrelor) in combination with aspirin in patients post ACS		I A
	P2Y_12_-receptor inhibitor continued for at least 12 months if ACS managed with PCI and stent placement		I A
Statins	Statin therapy indefinitely for all patients with a prior cardiovascular event.		I A
Angiotensin-system agent	ACE inhibitor (or ARB if intolerant) post-STEMI indefinitely for all patients with left ventricular ejection fraction <40% and in those with hypertension, diabetes, or chronic kidney disease		I A
	ACE inhibitor (or ARB) for all patients post-STEMI		IIa B
Beta-blockers	Beta-blockers for all patients post-STEMI		I A
	Beta-blockers continued for at least three years post-STEMI		I B

*Strength of Evidence.

I: Conditions for which there is evidence and/or general agreement that a given treatment is beneficial, useful, and effective.

IIa: Weight of evidence/opinion is in favor of usefulness/efficacy.

A: Data derived from multiple randomized clinical trials or meta-analyses.

B: Data derived from a single randomized trial, or nonrandomized studies.

Medication non-adherence is increasingly recognized as a very important issue due to its significant health consequences [19,20]. Many reasons for non-adherence have been proposed and these can generally be categorized as provider-level (*e.g.*, knowledge, motivation, time), patient-level (*e.g.*, knowledge, motivation, finances [21]), and system-level (*e.g.*, access to care, coordination of care). Furthermore, both ethnicity [22,23] and socio-economic status [24] seem to be related to quality of care for cardiovascular disease, even in countries with universal healthcare like Canada and the United Kingdom (UK), and non-adherence related to such factors may not be readily impacted with quality improvement interventions.

Fortunately, the evidence suggests that many of the key factors contributing to cardiac medication non-adherence may be amenable to intervention. Discontinuation of evidence-based cardiac medicines post-STEMI is rarely due to an active, informed choice after discussion of risks and benefits between patient and health-care-provider; absolute contraindications are rare and side effects are infrequently reported by patients as the primary reason for discontinuation (less than 4% of patients) [25,26]. In contrast to situations where informed decisions are made to deviate from standard treatment protocols, qualitative work in primary care has found that poor adherence may be frequently due to fragmented systems of care [27] or communication problems at the interface between secondary and primary care [28]. A recent study in Canada has highlighted the risk related to transitions in care; it appears that hospitalizations increase the risk for inadvertent discontinuation of cardiac medications [29].

The provider also can have an impact; having a cardiologist involved in the patient’s care may increase rates of appropriate medication adherence [30]. However, there is undesirable variation among prescription rates by specialists as well. In one study of cardiologists, the most common reason given for not prescribing secondary prevention medications was, ‘not high-enough risk’ [25]. However, in that study, risk scores of patients not treated for this reason were often higher than those of patients prescribed such treatment. Meanwhile, the same study found that approximately one-third of patients had stopped their medication without instruction from their doctor. This indicates a potential role for multi-pronged interventions addressing both the provider and the patient.

### **Previous research aiming to improve adherence**

Numerous systematic reviews have been published regarding interventions to improve adherence to medications. An overview of reviews found that no patient-mediated interventions were effective across all diseases, but found that the most promising interventions included self-management, simplified dosing, and involvement of pharmacists [31]. A review focusing on anti-depressants found that patient education alone was ineffective [32], and a review focusing on anti-epileptics found that patient education was inconsistent, while interventions with multiple reminders featuring action planning were more often effective [33]. Recognizing that non-adherence tends to worsen over time, a recent Cochrane review recommended testing a delayed intervention as opposed to the immediate reminders used in similar previous trials [34], as one would expect a larger effect size in a delayed intervention.

One previous trial has shown that brief evidence summaries regarding medications attached to discharge letters sent to primary care providers resulted in improved adherence [35]. Three other trials have evaluated the role of reminder letters to the primary care provider (with or without patient reminders) to improve adherence to evidenced-based cardiovascular therapies: one in the USA, one in the UK, and one in Canada [36-38]. The American trial focused on beta-blocker use post-MI and found a small increase in compliance (proportion of days covered), with a number needed to treat of 16 for achieving high adherence, but no change in the proportion who discontinued their beta-blocker. The other two trials focused on statin use in patients with known CAD. These trials found absolute increases in adherence of 9% to 10% in statin use, but despite this being a potentially important effect size on a population basis, both were under-powered for effects this size.

## Objectives

The overarching goal of this project is to improve long-term use of secondary prevention medications for patients with CAD, and thereby reduce cardiovascular events through the use of an easily generalizable and sustainable intervention. The primary objective of this study is to assess if repeated mailing of an educational message and reminder to the family physician and the patient will decrease the proportion of patients who discontinue evidence-based secondary-prevention medications at twelve months post-STEMI. A secondary objective is to encourage cardiac patients and their primary care providers to discuss the benefits of long-term adherence to cardiac medications.

## Methods/design

### **Study design**

**Figure 1:**
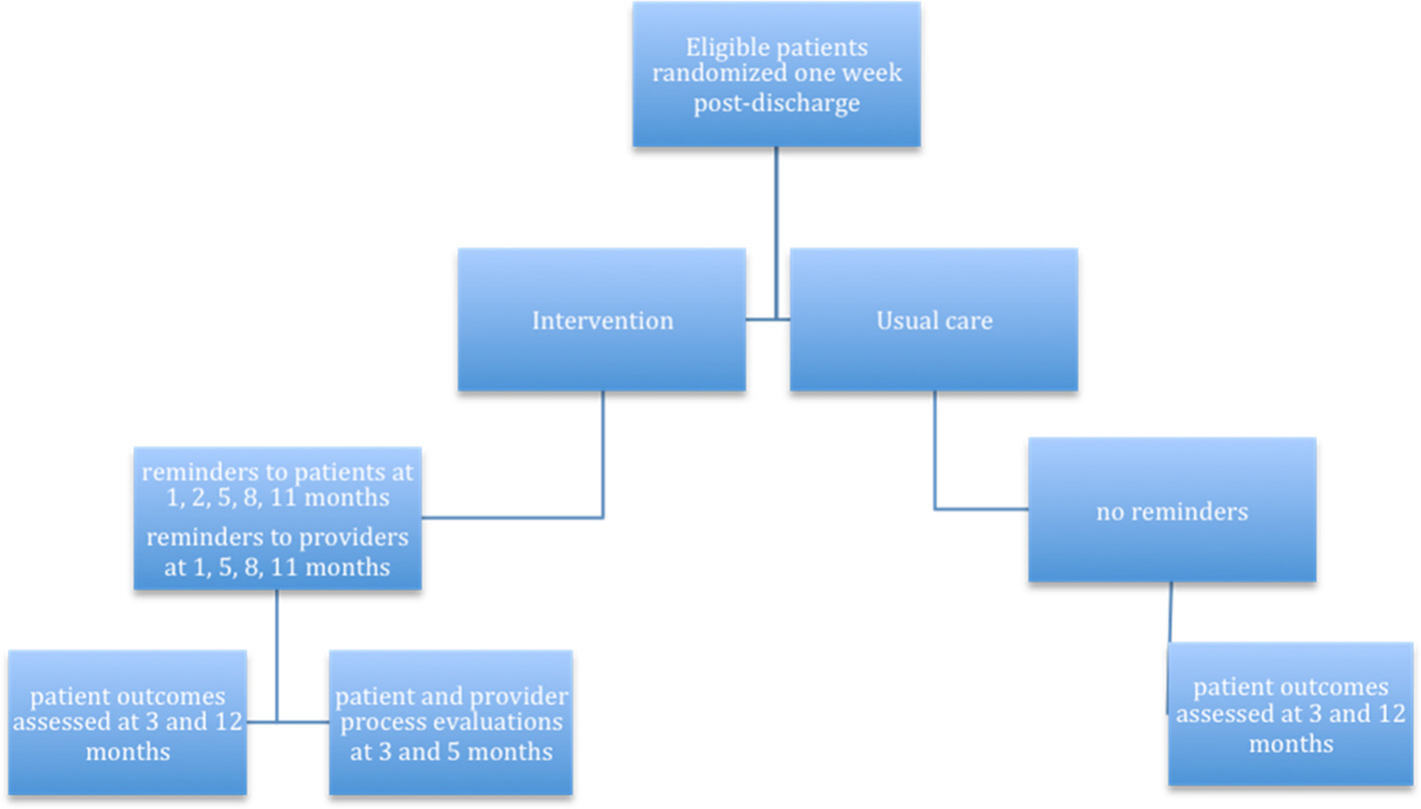
Study Flow Diagram.

DERLA-STEMI is a pragmatic, cluster-randomised controlled trial, with blinded outcome-assessment, and is registered with clinicaltrials.gov (NCT01325116). See Study Flow Diagram (Figure 1).

### **Participants and Setting**

In Ontario, healthcare is financed through a single-payer (publicly administered) system. There are no co-payments for visits to generalist or specialist physicians or for care provided in hospitals for patients of any age, and almost all licensed prescription medications are covered for patients 65 and over. Patients younger than 65 years pay for medications out-of-pocket or through private insurance plans, or are covered by the provincial plan if they qualify for social support.

In this study, eligible patients are adult patients (>18 years) with a diagnosis of STEMI, who undergo a coronary angiography procedure (with or without angioplasty), at the Heart Investigation Unit (HIU) in Hamilton, Ontario, and who are alive at hospital discharge. In keeping with the pragmatic approach to study design, no other exclusion criteria will be applied. The HIU is the only catheterization lab in its region, with a catchment population of almost 1.5 million people. More than 700 STEMI patients undergo an angiogram there each year. Studies at the HIU have highlighted excellent rates of prescribing of evidence-based therapies at discharge post-STEMI, but substantial reduction in use starting three months following discharge [39,40]. While 78% of STEMI patients leave the HIU taking a statin, an ACE-inhibitor (or ARB), a beta-blocker, and aspirin, by 90 days the proportion still taking all four of these medication classes falls to 63% (unpublished data from the Strategic Management of Acute Reperfusion and Therapies in Acute Myocadial Infarction (SMART-AMI) study).

### **Intervention**

The intervention was developed in concert with clinical experts from both primary care and cardiology, as well as experts in knowledge translation and medical decision-making. Personalized letters sent via post to the patient and their family physician at one, five, eight, and eleven months after their angiogram, signed by the interventional cardiologist (see Additional file 1: Appendix A for prototype). The letter for the family physician names the patient and provides brief evidence in support of long-term medication use for these patients. This was reviewed and edited with a series of family physicians from a different area of the province.

The patient letter provides a review of the importance and role of each of the cardiac medications and urges short- and long-term adherence (see Additional file 1: Appendix B for prototype). This educational aspect is designed to address knowledge and beliefs about medication use as a potential cause of poor adherence. The intervention explicitly encourages discussion of medication adherence with the family physician by asking patients to bring the letter to their family physician. It also asks patients to deliver the final page of their letter to their pharmacist; this page is written to the pharmacist urging them to participate in promoting long-term adherence. The intention is to facilitate recurrent discussions with primary care providers that emphasize long-term adherence and to address coordination of care and continuity of information as barriers to medication persistence. This letter was developed with iterative evaluations of understanding and acceptability amongst a series of cardiology patients at the HIU. The language in the patient letter is simplified to a grade six-level.

The timing of the intervention was specifically chosen based on the preliminary data obtained from the SMART-AMI trial demonstrating suboptimal rates at 90 days. Furthermore, literature (referenced above) demonstrates that adherence starts decreasing by thirty days and continues to decrease in an almost linear fashion. Finally, the common practice in Ontario is for pharmacists to dispense medications for no more than three months at a time (regardless of duration of the prescription ordered by the physician). Therefore, we decided to deliver the intervention at regular intervals (1, 5, 8, and 11 months post-STEMI) corresponding to the likely time periods prior to patients requiring a prescription renewal/refill. In pilot testing the intervention with family physicians and patients, we determined that sending the full letter too frequently would be undesirable and that the physicians in particular did not want to have monthly reminders. At the same time, close examination of data from Ontario indicated large stepwise declines in adherence at 30 and 60 days post-STEMI. To address this, patients will be provided an additional postcard type reminder two months post-STEMI (see Additional file 1: Appendix C).

In summary, the unique aspects of the intervention compared to usual care include the following: the letter to the primary care provider is personalized and includes a summary of the evidence in support of long-term adherence and represents a recurrent form of contact between the cardiologist and the primary care provider; the letter to the patient use clear language suitable for a broad range of health literacy levels and was iteratively refined with input from patients in the target population, features content that attempts to address adherence-related beliefs, and provides explicit, actionable instructions to discuss the matter with the family physician as well as a summary to be given to the outpatient pharmacist to facilitate coordination of renewals.

### **Comparator/usual care**

Usual care in this context may include some contact between the admitting physician (generally not the interventional cardiologist) for the STEMI patient and the primary care provider (generally the family physician). This is usually in the form of a standard discharge summary mailed to the family physician’s office at the end of the hospitalization. The quality of such discharge summaries varies widely even within the same institution (and summaries frequently lack necessary information regarding medications) [41]. In keeping with the pragmatic nature of the trial, no attempt will be made to standardize the usual care arm [42].

### **Allocation**

The randomization schedule was computer-generated by a statistician independent of the study, using a permuted block design with randomly varying block lengths of four, six, or eight. Eligible patients are randomly allocated to one of the two treatment arms. Although enrolment of more than one patient treated by a particular family physician is expected to occur infrequently, randomization will be carried out to ensure that, once a patient from any family physician is randomized, all future patients seen by that family physician will automatically be assigned to the same arm. This is necessary to avoid contamination (with one family physician having patients in different intervention arms). Roughly one-half of patients will be allocated to each study arm (the actual allocation ratio will depend on the size of the clusters). Based on pilot data, we anticipate that approximately 10% to 15% of patients will not have a family physician. In keeping with the pragmatic design of the trial, a patient without a family physician will be included (receiving only the patient-level intervention).

Randomization is delayed by one week (after the angiogram) to permit time to identify and exclude patients with in-hospital death. Randomization will continue until the target sample size is achieved. The anticipated duration of enrolment is 15 months. The allocation sequence will be concealed from the investigators and outcome assessors; only the study coordinator who will be sending out the letters will have access to the un-blinded allocation list.

### **Outcomes**

The primary outcome is the proportion of living patients who describe taking all cardiac medication classes of interest measured at twelve months. This type of ‘all-or-none’ measure has been recommended for evaluating quality improvement interventions, especially related to medication utilization [43]. Specifically, we will assess whether patients are taking a statin, beta-blocker, angiotensin modifier (ACE or ARB), and aspirin at twelve months. All STEMI patients have reasonable evidence supporting these medications [3]; we anticipate that randomization will balance those patients for whom evidence is less clear or who might have contraindications to any of these medications.

We will also assess whether patients are taking these four medication classes plus a secondary antiplatelet (clopidogrel, prasugrel, or ticagrelor) at three months. Therefore, patients at three months will be dichotomized as to whether or not they are taking all five cardiac medication classes, and at twelve months they will be dichotomized according to whether they are taking all four relevant medication classes. The difference in the number of medication classes considered at three and twelve months relates to uncertainty in the evidence regarding the appropriateness of a secondary antiplatelet at this timeframe. Additional secondary outcomes include a comparison of: the proportion of patients who report actively taking each cardiac medication class of interest (item-by-item) at three and twelve months; the proportion of patients who report stopping medications due to side effects at three and twelve months; the proportion taking one or two or three medication classes concurrently at three and twelve months; and the proportion of patients with a perfect Morisky Medication Adherence Score (MMAS) for cardiac medication compliance at three and twelve months. The MMAS is a brief, standardized adherence questionnaire which excellent reliability [44], and has been shown to be predictive of cardiovascular medication adherence [45] and to be associated with control of blood pressure and cholesterol [44,46]. In addition, all patients will be asked at three months and twelve months whether they had a discussion with their family physician during past three months in which the provider had encouraged long-term cardiac medication compliance.

### **Data collection**

Baseline patient characteristics will be obtained from standard patient-registry information at the HIU. This includes demographic information, comorbidities, and the findings at the time of angiography.

Outcomes will be assessed 3 and 12 months post-index angiogram through patient phone calls by a research coordinator associated with the HIU who will be trained expressly for this function. The research coordinator conducting the phone calls will not have access to the allocation list. The calls are made on behalf of the treating cardiologist at the HIU, and all patients will be encouraged to review cardio-protective meds with their family physician. The phone call follow-ups will ask patients to list their current, daily medications (and doses) without specific prompting in order to reduce bias. Attempts will be made to contact patients for a maximum of 30 days prior to being considered lost-to-follow-up. Reasons for loss-to-follow-up will be tracked.

For a sample of patients aged 65 and older, the Ontario Drug Benefit database will be used to examine the accuracy of the self-reported primary outcome and to further evaluate adherence using the medication possession ratio over the preceding year, which has been shown to be associated with both pill counts and clinical effects [47].

### **Ethical considerations**

Research Ethics Board (REB) approval was received at Hamilton Health Sciences Centre and McMaster University (project number 11–191). Given the low risk nature of the intervention, which falls within the realm of continuity-of-care and circle-of-care, the REB agreed that verbal consent at the time of outcome assessment is the most appropriate design to test this pragmatic intervention. Thus, there is no formal recruitment process; as mentioned above, all eligible patients within the registry at the HIU are allocated to intervention or control one-week post-STEMI. To gain REB approval, we agreed to provide a note to the family physician of all included patients describing the patient-reported outcomes (*e.g.*, current medications and adherence) at the end of the trial.

### **Data management**

All patient data will be collected directly into a password-protected database and will not be removed from the server at the HIU research office. Necessary information for contacting the participants (*e.g.*, name, phone number) will be kept in a separate, password-protected file from the study data, which will have no patient identifiers. The outcome data (without any identifiers) will be transferred from the database into a statistical package for analysis.

### **Analysis**

Descriptive statistics will be calculated for all variables of interest: continuous variables with a normal distribution will be summarized using means and standard deviations (medians and inter-quartile ranges in the case of skewed distributions), whereas categorical variables will be summarized using frequencies and proportions.

We hypothesize that the intervention will result in a greater proportion of patients who report taking each cardiovascular medication class of interest at 12 months post-angiography. The absolute difference in proportions will be calculated for all primary and secondary dichotomous outcomes, together with 95% confidence intervals adjusting for clustering by family physician [48]. The statistical significance of differences between arms will be evaluated using chi-squared tests, adjusted for clustering by family physician.

Exploratory multivariable analyses will be carried out using generalized estimating equations (GEE) to identify potential baseline predictors of adherence. Potential effect modification by treatment—medical management versus coronary artery bypass graft (CABG) versus angioplasty—and attendance at cardiac rehabilitation will be explored by including interactions between these two variables and group. It is plausible that this analysis will suggest a need for tailored interventions for these subgroups. A further exploratory analysis will be conducted focusing on patients who reported taking all five cardiac medication classes and had perfect MMAS scores at three months using a multivariable model to examine covariates predicting late-onset discontinuation. In addition, a planned sensitivity analysis will exclude those patients who did not have a family physician, as we would expect such patients to be more likely to discontinue their cardiac medications.

Analyses will be performed on an intention-to-treat basis. No interim analyses are planned. All analyses will be carried out using the SAS Version 9.2 statistical program (SAS Institute, Cary, NC, USA).

### **Sample size**

The sample size for this design is based on the following assumptions: an assumed absolute increase in the proportion of patients taking all four cardiovascular medication classes of 11% at twelve months post-STEMI; an estimated control group proportion of 50%, and a variance inflation factor of 1.02 (derived from an intra-cluster correlation coefficient of 0.019 calculated from data in the SMART-AMI registry and assuming an average cluster size of 1.2 based on pilot data). To achieve 80% power to detect a significant main effect of the intervention using a Chi-squared test at the 5% level of significance, 652 patients would be required. We will randomize 815 patients to account for an estimated participation rate of 80% at the 12-month follow-up. This dropout rate is conservative based on similar studies at the HIU where the participation rate has been greater than 90% over even longer time periods [40].

The expected effect size is slightly higher than the effect seen in the previous Canadian trial to account for the fact that the intervention is multifaceted (directed at both physician and patient) and occurring later post-STEMI (reducing the expected control group rate and therefore the possibility of a ceiling effect). Based on this sample size calculation, and the rate of STEMI patients presenting to the HIU, we anticipate that it will take approximately 15 months to complete the recruitment for this study.

We will use a kappa statistic to assess agreement between the self-report of the primary outcome and the corresponding objective data from the Ontario Drug Benefit database. Based on an anticipated overall proportion of 56% at 12 months (average of intervention and control arm) and an anticipated kappa of 0.88, we would consider acceptable agreement if the lower limit of the 95% confidence interval around kappa does not drop below 0.80. Therefore we will evaluate validity of the primary outcome in a random selection of 138 patients aged 65 or greater.

### **Process evaluation—optimizing the intervention**

A random sample of participating patients will be asked a series of additional, structured questions at the time of outcome assessment 90 days post-STEMI. Specifically, a 20% random selection of patients who received the intervention will be sampled, equating to approximately 80 patients. In addition, all family physicians in the intervention group will be mailed a one-page questionnaire along with the second iteration of the provider letter (month five post-STEMI). A response rate of only 15% will allow us to get feedback from about 50 family physicians. The questionnaires to both patient and provider assess acceptability of the intervention and the reasons for any (lack of) action taken (See Additional file 2: Appendix D for patient process evaluation questionnaire and Additional file 2: Appendix E for provider process evaluation questionnaire**)**. The answers to these questionnaires will be summarized descriptively and used to inform future iterations of the intervention.

We also plan to conduct focus groups with both patients and providers to better understand both why the intervention did (or did not) work and how it might be optimized. Participants for these focus groups will be purposively recruited based on the responses to the questionnaires. We plan to conduct one or two focus groups of six to eight patients and one focus group of four to six physicians, each group lasting about one hour occurring at the HIU. These focus groups will follow a semi-structured guide that will be informed by the issues identified in the questionnaires. The overarching goal of the focus groups will be to compare and contrast various designs and approaches of sending reminders to decrease the risk of inappropriate medication discontinuation. To this end, a variety of reminder designs will be handed out among the focus group participants to encourage discussion (similar to how marketing firms traditionally have used focus groups). Physician participants will be provided with $75 (and refreshments). Patient participants will be offered a $25 gift certificate (and refreshments) as remuneration for attending the focus group. The sessions will be recorded and transcribed verbatim.

## Discussion

Discontinuation of cardiac medications post-STEMI occurs due to patient, provider, and system-level factors and has important consequences for the patient. This two-arm, pragmatic, cluster-randomized controlled trial will test whether mailed reminder letters sent from the interventional cardiologist to the patient and their family physician can successfully increase adherence. Even if the trial does not show a significant effect on medication discontinuation, the embedded process evaluation will provide helpful information for planning future interventions aiming to address this important issue.

### **Limitations**

Although our overall goal is to improve adherence to medications, it is important to note that our primary outcome evaluates discontinuation (or ‘persistence’). This represents the most extreme form of non-adherence. The allocation is clustered at the level of the family physician to limit contamination, but it was deemed not feasible to do the same with pharmacists. Although the tear-away page in the patient letter for pharmacists could theoretically bias toward a null finding if pharmacists transfer their learning from one patient to another, we considered this risk to be small in comparison to the potential benefit of facilitating interactions with these key primary care providers.

It is important to note that this trial will not be able to discern the relative importance of intervention at patient versus family physician level. A larger sample size would be preferable to provide an opportunity to test multiple ways of designing and delivering this type of intervention within a single trial. In the case of a positive effect, the pragmatic approach utilized will not allow for inferences regarding the ‘most important’ active ingredients in the intervention. We intend to explore these issues through the process evaluation. The questionnaires developed for the process evaluation are not independently validated for assessing acceptability and usability of the reminder intervention. However, they will be evaluated qualitatively to inform iterative improvements to the program after the trial is completed, and will allow us to identify interested participants for focus groups.

Although the research coordinator conducting the outcome assessment will be blinded to allocation, it is possible that some patients will discuss receiving the intervention with the coordinator. Another important caveat is that we will be using patient self-report for main outcome measurements—an approach which has previously been used in a similar trial [38]. We are planning to evaluate the validity of self-report data in our study by comparing patient reported medication use (including the MMAS) with data recorded in administrative databases for a subsample of participants above age 65 (for whom data are accessible using the Ontario Drug Database). Through these administrative databases, we can also pursue proxy measures for adherence (rather than strictly discontinuation) by assessing the medication possession ratio.

### **Implications**

Given the proven effectiveness of secondary prevention medications for reducing morbidity and mortality and the high risk for poor outcomes in the post-STEMI population, we believe it is appropriate to power this trial to show relatively small increases in adherence. Although we would hesitate to extrapolate the findings of this study without further research, we believe it is important for quality improvement trials measuring process outcomes such as adherence to consider the potential for patient-relevant outcomes. To illustrate, consider the systematic review of RCTs of statin-therapy, which found a number needed to treat (NNT) of 86 to reduce mortality in patients with CAD [49]. We estimate that the NNT for avoiding statin discontinuation with the reminder interventions tested in this trial is approximately 10. If this were the case, then the NNT for the reminder interventions to prevent a single mortality would be 860. In fact, the number might be lower than this since the intervention may also increase utilization of the other cardiac medications known to reduce mortality. Given the low-cost, low-risk nature of the intervention in this trial, we believe that NNTs of this size merit further study for potential population-wide implementation.

### **Summary**

The major strengths of this trial are the pragmatic nature of the intervention the study design. The trial is well powered and designed specifically to improve health services for a common problem in patients at high risk of cardiovascular events. Many quality improvement trials embark upon highly sophisticated and expensive interventions; even if successful, sustainability of such interventions beyond the trial period proves challenging. Conversely, the DERLA-STEMI intervention would be easily testable in other healthcare settings and for other conditions where long-term adherence is suboptimal. We believe this study will demonstrate the feasibility, acceptability, and (hopefully) the effectiveness of a sustainable and generalizable quality improvement intervention for STEMI patients.

## Competing interests

The authors declare no conflicts of interest.

## Authors’ contributions

All authors contributed to the study concept and design and all approved the final version of this manuscript.

## Supplementary Material

Additional file 1 **Appendix A.** Physician Intervention Letter. **Appendix B.** Patient Intervention Letter. **Appendix C.** Patient Intervention 2:-month Postcard. Click here for file

Additional file 2 **Appendix D.** Process Evaluation, patient survey. **Appendix E.** Process Evaluation, provider survey. Click here for file
