# Supplementary material for: Delayed educational reminders for long-term medication adherence in ST-elevation myocardial infarction (DERLA-STEMI): Protocol for a pragmatic, cluster-randomized controlled trial
Source: Implement Sci. 2012 Jun 9;7:54. doi: 10.1186/1748-5908-7-54 (PMC3536678; doi:10.1186/1748-5908-7-54)
Supplement: Additional file 1 — Appendix A. Physician Intervention Letter. Appendix B. Patient Intervention Letter. Appendix C. Patient Intervention 2:-month Postcard. [file 1748-5908-7-54-S1.docx]

**Appendix A. Physician Intervention Letter**

**[DD-MMM-YYYY]**

Dear Dr. **[FAMILY PHYSICIAN NAME]**

RE: Our patient with coronary artery disease: **[PATIENT NAME, DOB: DD-MMM-YYYY]** (*If this is not your patient, please let us know as soon as possible)*

In **[MONTH, YYYY]**, this patient was admitted for a myocardial infarction (STEMI) and had a coronary angiogram at the Hamilton General Hospital Heart Investigation Unit.

As the family physician, we believe that **you are in the best position to help this individual adhere to the medications and lifestyle changes that can improve prognosis.**

Optimal cardiac medication management has been shown to reduce cardiovascular disease events in patients like **[PATIENT NAME]** by 75-80%.^1,2^ For most post-MI patients, optimal medical management includes long-term use of a **statin, aspirin, clopidogrel, beta-blockers, and an ace-inhibitor**.^3^ However, research has shown that by one year post-MI, between one third and one half of patients are non-adherent.^4^

On the next page, we include a summary of the general recommendations for medication-based management of cardiovascular risk. **We acknowledge that preventive medications must be understood as a part of this patient’s overall health and wellbeing**.

We have mailed a similar letter directly to **[PATIENT NAME]** to remind them of the importance of their cardiac medications and may send further reminders. If you have any questions or concerns about these letters or the medication recommendations for this patient, please don’t hesitate to contact us.

Please **consider assessing (and encouraging) medication adherence during your next visit** with this patient.

Sincerely,

The cardiologists at the Heart Investigation Unit

Hamilton Health Sciences, General Site

237 Barton Street East, Hamilton, ON, L8L 2X2

Phone: 905-577-1423, Fax: 905-577-1474

**Medical Management post-STEMI: Combination of 5 medications**

- **(1) Aspirin and (2) clopidogrel** constitute the mainstay of antiplatelet therapy post-MI.
  - Aspirin therapy (75-162 mg/day) **should be continued indefinitely** post-STEMI (IA).
  - Clopidogrel is recommended for a minimum of 14 days in patients not undergoing percutaneous coronary intervention (PCI) post-STEMI (IB), but **ideally for 1 year**. Clopidogrel is recommended for a minimum of 1 month and ideally 1 year in patients post PCI with a bare-metal stent (IB) and a minimum of 1 year post PCI with a drug-eluting stent (IB).
    - An alternative to clopidogrel is prasugrel or ticagrelor
- **(3)** A **Statin** should be initiated in hospital, regardless of baseline lipid status or dietary modification and **continued indefinitely**, for all patients, in the absence of contraindications, with a prior myocardial infarction (IA).
  - Early intensive statin therapy is recommended for reducing recurrent cardiovascular events (e.g. atorvastatin 80mg or simvastatin 40 mg).^5,6^
  - Intolerance to a statin may be alleviated by switching to pravastatin or fluvastatin.^7^
- **(4)** An **ACE-inhibitor (ACEI)** should be initiated and **continued indefinitely** for all patients post-STEMI with reduced left ventricular ejection fraction (LVEF < 40%), hypertension, diabetes or chronic kidney disease (IA).
  - Most other STEMI patients will also benefit from and ACEI or ARB (IB).
  - An alternative to ACEI is an angiotensin receptor blocker.
- **(5) Beta-blockers** should be initiated and **continued indefinitely** in all patients post-STEMI (IA).

**References:**

^1^Yusuf S. Two decades of progress in preventing vascular disease. Lancet. 2002;360:2–3.

^2^ Wald NJ, Law MR. A strategy to reduce cardiovascular disease by more than 80%. BMJ. 2003;326:1419–1424.

^3^ The Canadian Cardiovascular Society and the American College of Cardiology support the initiation and maintenance of pharmacotherapy including aspirin, clopidogrel, statin, beta-blocker, and angiotensin blockers with Class I support and Grade A or B evidence.

^4^ Newby LK, LaPointe NM, Chen AY, Kramer JM, Hammill BG, DeLong ER, Muhlbaier LH, Califf RM. Long-term adherence to evidence-based secondary prevention therapies in coronary artery disease. Circulation. 2006;113(2):203-12.

^5^ Cannon CP, Braunwald E, McCabe CH, et al. Intensive versus moderate lipid lowering with statins after acute coronary syndromes. N Engl J Med 2004;350:1495–504.

^6^ de Lemos JA, Blazing MA, Wiviott SD, et al. Early intensive vs a delayed conservative simvastatin strategy in patients with acute coronary syndromes: phase Z of the A to Z trial. JAMA 2004;292:1307–16.

^7^ Graham DJ, Staffa JA, Shatin D, et al. Incidence of hospitalized rhabdomyolysis in patients treated with lipid-lowering drugs. JAMA 2004; 292:2585.

**B. Patient Intervention Letter**

PERSONAL AND CONFIDENTIAL FOR **[PATIENT NAME]**

If you are not John Doe, please tell us immediately by calling 905-527-0271, Ext 44757.

Date: **DD-MMM-YYYY**

Dear Mr. **[PATIENT LAST NAME],**

In **[MONTH, YEAR]**, we cared for you in our hospital after you had a heart attack. A heart attack can be a very difficult event. We hope you are feeling better these days.

As you may already know, once you have had a heart attack, you have a higher risk of having another heart attack or a stroke. Fortunately, there are ways you can lower your risk of future heart attacks and strokes.

Even if your lifestyle is very healthy, scientific research has clearly shown that **taking the right** **pills will lower your risk** of future heart attacks and strokes.

# Make sure you are taking the right pills

Please **bring this letter and all your pills with you** **to your next appointment with your family doctor and heart doctor (also called a cardiologist)**.

Talk to your family doctor and heart doctor about the 5 kinds of pills listed in this box. The same kind of pill can be called by different names. Make sure you are taking the right pills for you.

National medical guidelines strongly recommend that patients who have had a heart attack take these 5 pills for a long time. For most of the pills, a long time means for the rest of your life.

- Cholesterol Pills

1. Cholesterol pills are called ‘statins’. A statin keeps cholesterol from building up on the inside of the walls of your blood vessels. Taking a statin is important because it will significantly lower your risk of future heart attacks or strokes, even if your cholesterol number is already low.

(continued on the next page …)

- Blood Thinners (both of the following):

2. Aspirin prevents heart attacks and strokes by making your blood less ‘sticky’.

*and*

3. Clopidogrel (Plavix) also makes your blood less sticky, especially in patients who have had an ‘angio*plasty*’ (balloon and stent to open the artery and help keep it open.) Unlike the other pills, this pill may be taken for as little as one month or up two years, depending on your heart doctor’s recommendations.

- Blood Pressure Medications (both of the following)

4. An ‘ACE Inhibitor’ or **‘**ARB**’** has a special, protective effect for your heart. It lowers your blood pressure and also does other things that help keep your heart healthy.

*and*

5. A ‘beta-blocker**’** protects your heart by making sure that it does not beat too fast or work too hard.

It is important to take your heart pills every day. Taking your pills as directed will make you less likely to have heart attacks and strokes in the future.

Most people who have had a heart attack should take most of these pills for the rest of their lives. However, some people have important reasons to stop or never take these medications, including other medical conditions, side effects, or allergies. For these people, other pills may be available.

# Talk to your doctor about your pills

It is important to talk about all of your medications with your doctor **on a regular basis.**

# Lifestyle changes help, too

In addition to taking your heart pills, you can also lower your risk of future heart attacks and strokes by living a healthy lifestyle. If you are a smoker, you need to try to quit. If you have trouble quitting, cutting back is better than nothing. Exercising at least 4 times each week and eating a heart-healthy diet will also help your heart.

See the next page for groups that can help you live a healthy lifestyle. You can also ask your doctor to refer you to a program called ‘cardiac rehab’ to help you make these changes.

Even if you live a healthy lifestyle, it is still very important that you keep taking your pills. Your best chance of staying healthy happens when you live a healthy lifestyle and take the right pills.

# Keep your heart as healthy as possible

Since we cared for you while you were admitted with a heart attack, we feel it is very important to remind you about how to lower your risk of future heart attacks and strokes.

Taking the right pills and living a healthy lifestyle will help keep your heart as healthy as possible, for as long as possible.

Sincerely,

Dr. J-D Schwalm on behalf of the cardiologists at the Heart Investigation Unit

Hamilton Health Sciences, General Site

237 Barton Street East

Hamilton, ON, L8L 2X2

Phone: 905-577-1423

Fax: 905-577-1474

P.S. While waiting for your next appointment with your doctor, here are some other ways you can get information:

Quitting smoking:

**Canadian Cancer Society Smokers’ Helpline**

Website: www.smokershelpline.ca

Telephone: 1-877-513-5333

A heart-healthy diet:

**Heart and Stroke Foundation**

Website: www.HeartandStroke.com/Healthy_Recipes
Telephone: (905) 574-4105

**Mayo Clinic**

Website: http://www.mayoclinic.com/health/mediterranean-diet/CL00011

Coronary artery disease:

**Heart and Stroke Foundation**

Website: www.HeartandStroke.com/Coronary_Disease

Telephone: (905) 574-4105

Cardiac rehab:

**Hamilton Health Sciences:** Telephone: (905) 577-8033

**St Catherines/Niagara:** Telephone: (905) 641-2542

**Brantford:** Telephone: (519) 751-5523

# Your pharmacist can help you make sure you are taking the right pills

It is important to **use the same pharmacy every time you get your pills**. When you always use the same pharmacy, the pharmacist can help you keep track of your medications.

**Please take this page to your pharmacist**. Pharmacists play an important role in your health care and can help you and your doctor make sure you are taking the right pills.

Dear Pharmacist of **[PATIENT NAME]**,

Date: **[DD-MMM-YYYY]**

This is a personal and confidential note regarding **[PATIENT NAME]**.

As you may be aware, **[PATIENT NAME]** recently had a heart attack. It is recommended that such patients take a statin, aspirin, ace-inhibitor, and beta-blocker indefinitely. The recommended length of time for clopidogrel varies from one month to two years. These medications have been shown to significantly reduce the risk of future heart attacks and strokes.

Unfortunately, many people stop their medications too early.

The purpose of this letter is to ask for your help to support **[PATIENT NAME]** in continuing to use these medications. Please, if possible, notify the patient’s family physician, Dr. **[FAMILY PHYSICIAN NAME]** when a renewal is needed. In addition, please contact **[PATIENT NAME]** when it is time for a refill. Finally, please encourage this patient to continue to take these medications as prescribed.

By alerting the family physician about any side effects and discussing alternative medications within the same drug class, it may be possible to improve long-term adherence. With your help, we can reduce **[PATIENT NAME]**’s risk of future heart attacks and strokes.

Sincerely,

Dr. J-D Schwalm on behalf of the cardiologists at the Heart Investigation Unit

Hamilton Health Sciences, General Site

237 Barton Street East

Hamilton, ON, L8L 2X2

Phone: 905-577-1423

Fax: 905-577-1474

**Appendix C. Patient Intervention 2-month Postcard**

Personal and Confidential for: **John Doe**

If you are not **John Doe**, please call immediately:
905-527-0271, Ext 44757.

We sent you a letter a month ago about how **taking the right pills can protect your heart**.

Most people who have a heart attack should take 5 kinds of pills each day for a long time: *Blood thinners* (usually aspirin combined with either clopidogrel, prasugrel, or ticagrelor), cholesterol pill (s*tatin)*, and blood pressure pills (b*eta-blocker, and* *ACE-inhibitor or ARB*).

Bring your pills to your doctor and **make sure you are taking the right pills** for your heart.

In case you did not get our letter last month, we have included a copy in this envelope.

Sincerely,

Dr. J-D Schwalm on behalf of the cardiologists at

the Heart Investigation Unit,

Hamilton Health Sciences
