# Supplementary material for: Delayed educational reminders for long-term medication adherence in ST-elevation myocardial infarction (DERLA-STEMI): Protocol for a pragmatic, cluster-randomized controlled trial
Source: Implement Sci. 2012 Jun 9;7:54. doi: 10.1186/1748-5908-7-54 (PMC3536678; doi:10.1186/1748-5908-7-54)
Supplement: Additional file 2 — Appendix D. Process Evaluation, patient survey. Appendix E. Process Evaluation, provider survey. [file 1748-5908-7-54-S2.docx]

**D – Process Evaluation, patient survey**

| Patient ID: | CTR: | Pt Screen #: |
| --- | --- | --- |

These questions should be added to the 90 day follow-up calls in patients identified to be randomized to the process evaluation.

Questions:

Did you receive a mailed letter from the cardiologists who did your angiogram?

Y	N

**[if no, then stop here]**

Did you read the letter?

Y	N

Was the letter written in a way that made it easy for you to understand?

Y	N

Did the letter talk about things that matter to you?

Y	N

Did you take the letter to your family doctor?

Y	N

Did you discuss the letter or the cardiac medications with any other health care providers (including pharmacists)?

Y	N

Is a letter like this an acceptable way to improve communication between the hospital and patients?

Y	N

Would you have preferred e-mail?

Y	N

Would you have preferred fax?

Y	N

Did the letter help you remember to take your cardiac medications?

Y	N

Did the letter help you remember to get your cardiac medications renewed?

Y	N

How could the letter be more helpful?

[Note main points]

Did you give the note to your pharmacist?

Y	N

If Yes, can you give us the pharmacy name and number?

Name:

Phone #:

**E – Process Evaluation, provider survey**

**[DD-MMM-YYYY]**

Dear Dr. **[FAMILY PHYSICIAN NAME]**

RE: Our patient with coronary artery disease: **[PATIENT NAME, DOB: DD-MMM-YYYY]** (*If this is not your patient, please let us know as soon as possible)*

We, the cardiologists of the Heart Investigation Unit at Hamilton Health Sciences, have initiated a project aimed at improving long-term use of secondary prevention medications for patients with coronary artery disease and thereby reducing cardiovascular events through the use of an easily generalizable and sustainable intervention. We were hoping you could help us to learn how we might improve this program by completing this 10 question survey regarding a letter you may have received approximately 2 months ago. Please circle the most correct statements below.

1. Do you remember receiving a similar letter, to the one enclosed herein, around 2 months ago?

**No	Yes**

2. Did your patient bring the letter they received from us to the office to discuss it with you?

**No	Yes**

The following questions are about **the letter we sent directly to you**.

3. The letter was helpful for encouraging a discussion of cardiac medications with my patient.

**Strongly Disagree	Disagree	Unsure	Agree	Strongly Agree**

4. The message in the letter was clear.

**Strongly Disagree	Disagree	Unsure	Agree	Strongly Agree**

5. The recommendations in the letter were applicable for the patient.

**Strongly Disagree	Disagree	Unsure	Agree	Strongly Agree**

6. Letters like this are an acceptable way for us to communicate with you.

**Strongly Disagree	Disagree	Unsure	Agree	Strongly Agree**

7. If this letter continues to be sent, what would be your preferred method?

**Postal mail	Fax	Email**

8. How could letters like this be more helpful?

9. Do you have other suggestions or ideas about how hospital-based specialists could play a role to increase long-term cardiac medication adherence?

10. We hope to improve this program; could we contact you to discuss this further?

**No	Yes**

**WE APPRECIATE YOUR HELP. ALL RESPONSES WILL REMAIN ANONYMOUS.**

**PLEASE FAX THE COMPLETED SURVEY BACK TO 905-526-0678**

Sincerely,

The cardiologists at the Heart Investigation Unit

Hamilton Health Sciences, General Site

237 Barton Street East, Hamilton, ON, L8L 2X2

Phone: 905-577-1423, Fax: 905-577-1474
